# Supplementary material for: PUMA gene delivery to synoviocytes reduces inflammation and degeneration of arthritic joints
Source: Nat Commun. 2017 Jul 27;8:146. doi: 10.1038/s41467-017-00142-1 (PMC5529536; doi:10.1038/s41467-017-00142-1)
Supplement: Supplementary file 1 — Supplementary Information [file 41467_2017_142_MOESM1_ESM.pdf]

File name: Supplementary Information
Description: Supplementary figure.

File name: Supplementary Movie 1
Description: The movie shows the reduced mobility, joint inflammation and overall behaviour of a rat treated with HAdV5-PUMA, at Day 21 post-treatment.

File name: Supplementary Movie 2
Description: The Movie 2 shows the normal mobility, reduced joint inflammation and overall behaviour of a rat treated with BVCARHAdV5-PUMA, at Day 21 post-treatment.

File name: Peer review file
Description:

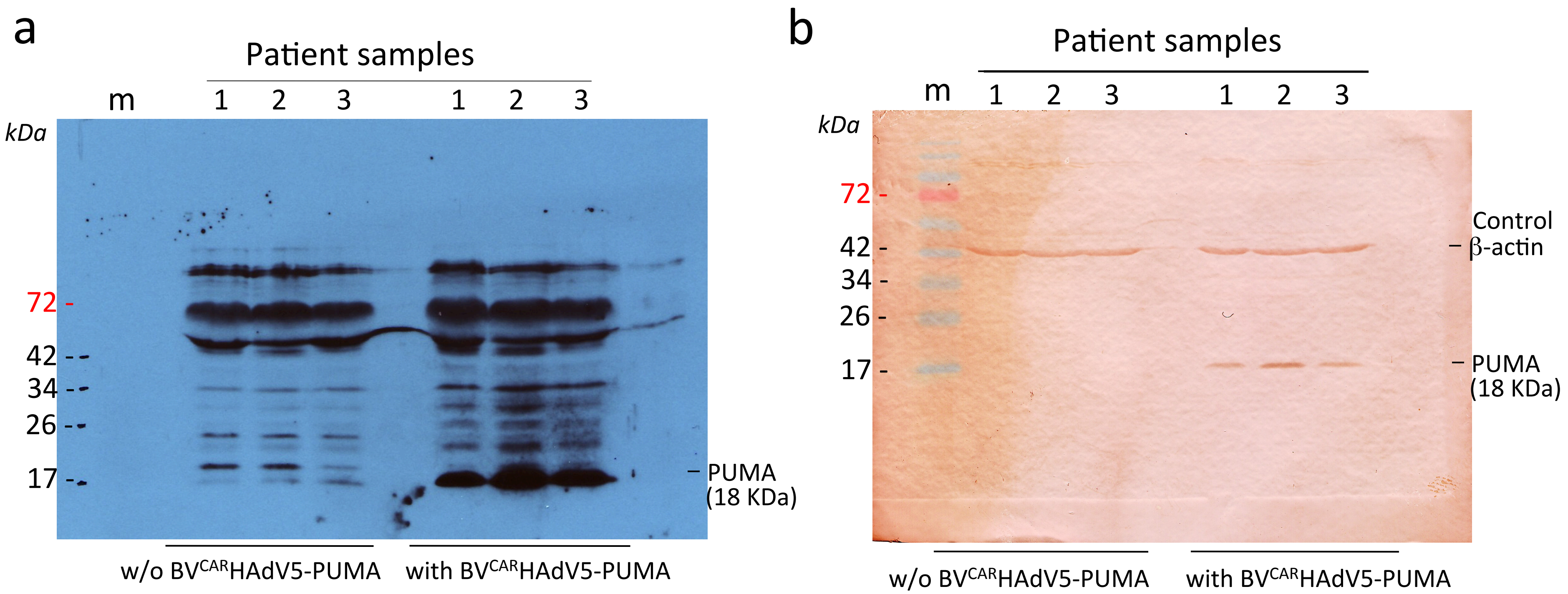

Supplementary Fig. 1. Original Western blot of PUMA protein expression in FLS, shown in Fig. 3 d. FLS derived from synovium explants of patients with RA were transduced or not with BV^{CAR}HAdV5-PUMA at 50 vp/cell. Cells were harvested at 40h post-transduction, lysed, and whole cell lysates probed for PUMA protein. ***a,*** Luminogram of transfer membrane reacted with anti-PUMA RabMAb, peroxidase-conjugated anti-rabbit IgG antibody and ECL Detection Kit. ***b,*** Same membrane reacted with anti-β-actin MoMAb, peroxidase-conjugated anti-mouse IgG antibody, and staining reaction performed with H_20_2 and 3,3'-Diaminobenzidine.
